# Factors Associated with Hepatitis B and C Co-Infection among HIV-Infected Patients in Singapore, 2006–2017

**DOI:** 10.3390/tropicalmed4020087

**Published:** 2019-05-27

**Authors:** Chiaw Yee Choy, Li Wei Ang, Oon Tek Ng, Yee Sin Leo, Chen Seong Wong

**Affiliations:** 1 National Centre for Infectious Diseases, 16 Jalan Tan Tock Seng, Singapore 308422, Singapore; chiawyee.choy@mohh.com.sg (C.Y.C.); li_wei_ang@ncid.sg (L.W.A.); oon_tek_ng@ttsh.com.sg (O.T.N.); yee_sin_leo@ncid.sg (Y.S.L.); 2 Department of Infectious Diseases, Tan Tock Seng Hospital, 11 Jalan Tan Tock Seng, Singapore 308433, Singapore; 3 Public Health Group, Ministry of Health, Singapore, College of Medicine Building, 16 College Road, Singapore 169854, Singapore; 4 Lee Kong Chian School of Medicine, Nanyang Technological University, 11 Mandalay Road, Singapore 308232, Singapore; 5 Yong Loo Lin School of Medicine, National University of Singapore, 10 Medical Drive, Singapore 117597, Singapore

**Keywords:** HIV, hepatitis, HBV, HCV, co-infection, prevalence

## Abstract

Co-infection of hepatitis B virus (HBV) and hepatitis C virus (HCV) with human immunodeficiency virus (HIV) is associated with increased risk of hepatic complications and mortality. A retrospective study to estimate the proportion of HBV and HCV co-infections in Singapore was conducted using a clinical database. We included 3065 patients who were seen under the Clinical HIV Programme at the largest referral centre for HIV care between 2006 and 2017 and were tested for both HBV and HCV. Factors associated with HIV-HBV and HIV-HCV co-infections were determined using logistic regressions. The majority (86.3%) of HIV-infected patients were mono-infected, while 7.2% were co-infected with HBV, 6.0% with HCV, and 0.5% were co-infected with both HBV and HCV. The most common HCV genotype was GT1 (63%). Factors significantly associated with HBV co-infection in the multivariable model were: Aged 30–49 years and 50–69 years at HIV diagnosis, male gender, and HIV transmission through intravenous drug use (IDU). Independent factors associated with HCV co-infection were: Malay ethnicity, HIV transmission through IDU, and HIV diagnosis between 2006 and 2008. Behavioural risk factors such as IDU, as well as epidemiologic differences associated with co-infection, should inform further studies and interventions aimed at reducing viral hepatitis infection among HIV-infected individuals.

## 1. Introduction

Human immunodeficiency virus (HIV), hepatitis B virus (HBV), and hepatitis C virus (HCV) are the most common chronic viral infections worldwide [1]. These three viruses share common modes of transmission, which include sexual transmission, sharing of needles with intravenous drug use (IDU), and transfusion of blood and blood products. Patients can often be co-infected with two, and even all three, viruses. 

Prior to the availability of highly active antiretroviral therapy (HAART), patients with HIV-HBV co-infection were 19 times more likely than patients mono-infected with HBV to die from liver-related complications [2]. The advent of tenofovir, lamivudine, and emtricitabine have led to significant improvements in reducing hepatic complications and related mortality. However, despite these advances, some studies continue to show that overall mortality and risk of hepatocellular carcinoma remains elevated in HIV-HBV co-infected populations compared to HIV or HBV mono-infected populations [3,4], while others have shown better prognosis with the advent of HAART [5].

Approximately 37 million people worldwide are infected with HIV, and about 7.4% of them are co-infected with HBV [6]. The estimated prevalence of HBV infection worldwide varies across different geographical regions [7]. In Singapore, the prevalence of HBV was 0.3% in children and adolescents aged 1–17 years between 2008 and 2010 [8], and 3.6% in adults aged 18–79 years in 2010 [9]. The reduction in the incidence of acute hepatitis B and the prevalence of chronic hepatitis B carriage in Singapore has been attributed to the introduction of hepatitis B vaccination into the national childhood immunisation programme (NCIP) in 1987 [10]. 

The estimated prevalence of HCV infection worldwide is 2.2% [11], while the estimated global prevalence of HCV co-infection among people living with HIV is 6.2% [12]. As of 2016, the prevalence of HCV in Singapore is estimated to be about 0.1% based on blood donor screening data and notifications of new HCV cases to the Ministry of Health (MOH) [13]. 

In Singapore, about 450 new cases of HIV are reported every year, and this number has remained fairly constant since 2008, with notification rates of 103.7 to 125.2 per million resident population [14]. The majority (97%) of newly-reported HIV infections from 2008–2017 were acquired through sexual transmission [14]. 

A local study investigating the risk factors and time trends for co-infections in a random sample of over 700 HIV-infected patients who were referred for first-time care from 2006 to 2011 found that 8.1% were co-infected with HBV and 2% had active HCV co-infection [15]. It is important to understand risks contributing to co-infection to guide public health interventions and surveillance. In this retrospective study, we investigated the epidemiological factors associated with HBV and HCV co-infection among HIV-infected patients in Singapore. 

## 2. Materials and Methods 

### 2.1. Study Population

The Clinical HIV Programme at the National Centre for Infectious Diseases (NCID) sees the highest number of HIV-infected patients in Singapore. The NCID is a 330-bed purpose-built facility and houses clinical services, public health, research, training, and education under one overarching structure. We made use of a clinical database maintained by the programme for the retrospective study, with the following information being routinely collected for all patients referred for care: Demographics, virologic and immunologic parameters, antiretroviral therapy (ART) and monitoring, and routine biochemical test results performed in the course of HIV care. 

The study population comprised Singapore residents who were diagnosed with HIV and attended the NCID at least once between 2006 and 2017. We confined the study subjects to HIV-infected patients who were tested for both HBV and HCV. HIV infection was confirmed by western blot assay following a positive result from a fourth-generation chemiluminescent microparticle immunoassay (CMIA). HBV infection was ascertained based on seropositivity for the hepatitis B antigen surface (HBsAg) using electrochemiluminescence immunoassay (ECLIA), and hepatitis B e-antigen (HBeAg) was also measured using ECLIA. HCV infection was based on seropositivity for the anti-HCV antibody (anti-HCV) test using ECLIA, or detectable HCV with real-time polymerase chain reaction (PCR). HCV genotyping was done by reverse line probe hybridisation.

Co-infection means an affected person is living with two or more infections at the same time. Individuals should not be considered as having both HIV and HBV and/or HCV infections simultaneously at any time point if they have been cleared of HBV or HCV prior to their HIV diagnosis. Approximately 95% of adults newly infected with HBV recover completely and do not become chronically infected [16,17]. After acute HCV infection, up to 45% of individuals achieve spontaneous viral clearance within six months of infection without any treatment, and do not progress to chronic infection [18,19]. Hence, patients with positive results for HBV based on HBsAg and/or HCV based on anti-HCV or HCV PCR, more than one year before being tested positive for HIV, were excluded, as a large number of these patients may have been cleared of HBV and/or HCV prior to their HIV diagnosis. We checked the subsequent test results of patients excluded in this way, and they were found to be negative for HBV/HCV. In addition, we would like to investigate risk factors associated with HIV and HBV/HCV co-infections. HBV or HCV infection acquired before HIV infection may have been due to risk factors that are different from HIV infection, hence they were excluded from the data analysis. Serologic test results up to the end of September 2018 were retrieved from the clinical database. Co-infection with HBV was defined as a positive test for HBsAg within one year before HIV diagnosis, or a positive HBsAg result after HIV diagnosis. Co-infection with HCV was defined as a positive test for anti-HCV and/or HCV PCR within one year before HIV diagnosis, or a positive HCV test after HIV diagnosis.

Epidemiological characteristics and laboratory parameters were compared in four groups of HIV-infected patients: HIV mono-infection, co-infections with HBV only, co-infections with HCV only, and HIV-HBV-HCV co-infections.

### 2.2. Statistical Methods

We calculated the 95% confidence intervals (CIs) for binomial proportions using Wilson’s method. The Chi-square test was used to test for group differences. Proportions between two groups were compared using two-sample independent z tests, with standard error estimated using pooled value of the two proportions. The Mantel-Haenszel linear-by-linear association Chi-square test was used to evaluate whether there was linear trend in proportion of co-infections over time.

The main outcomes of interest were whether the patient had HBV or HCV co-infections or not. For univariable logistic regression analyses, odds ratios (OR) and 95% CIs were calculated. Multivariable logistic regression was used to determine independent risk factors associated with co-infections, and adjusted odds ratios (aOR) were calculated. Variables were considered for inclusion in the multivariable model using the likelihood-ratio statistic based on the maximum partial likelihood estimates in backward stepwise selection with *p* > 0.10 for removal of variables. All variables with *p* < 0.05 were retained in the final multivariable model.

The Kaplan-Meier method was used to estimate the five-year survival rate. All reports of deaths, irrespective of cause, which occurred by the end of 2017 were included in the survival analysis. Patients contributed person-time from the date of HIV diagnosis until death or 31 December 2017, whichever was the earlier date. 

All *p* values reported were two-sided, and statistical significance was taken as *p* < 0.05. Statistical analyses were performed using the IBM SPSS Statistics for Windows, version 24 (IBM Corp., Armonk, NY, USA).

### 2.3. Ethical Considerations

Ethical approval for use of the clinical database was obtained from the Singapore National Healthcare Group Domain Specific Review Board (NHG DSRB reference number 2012/00438). Informed consent was not obtained as the clinical data collected was used as part of the care management of HIV patients. All data analysed for the study were anonymised. 

## 3. Results

A total of 3327 HIV-infected patients were seen under the Clinical HIV Programme from 2006 to 2017. There were 3139 patients who had been tested for HBV, and among them, 3097 had also been tested for HCV. After excluding 32 who had been tested positive for HBV and/or HCV more than one year before HIV diagnosis and subsequently tested negative, a total of 3065 HIV-infected patients constituted the final sample for this study. The proportion of HBV co-infection among HIV-infected patients was 7.7% (95% CI: 6.8–8.7%), while the proportion with HCV co-infection was 6.6% (95% CI: 5.7–7.5%). The majority (86.3%) were mono-infected, while 0.5% were co-infected with HBV and HCV. 

The mean age at HIV diagnosis was 40.2 years (standard deviation 12.7; range: 13 to 88). Adults aged 20–49 years at HIV diagnosis comprised about 74.3% of the study sample. The majority (93.8%) were men, and over three-quarters (76.0%) were Chinese. The main mode of HIV transmission was via sexual exposure (96.6%). Most of the HIV-infected patients (95.5%) were on antiretroviral treatment at the time of analysis.

Patients with triple infections of HIV, HBV, and HCV were diagnosed with HIV at a younger age (mean age 36.8, median age 35); the majority (87.6%) were aged 20–49 years at diagnosis (Table 1). About three-quarters of patients with HIV mono-infection, and those co-infected with HCV only, were in the age group of 20–49 years; 73.8% in HIV mono-infected patients (mean age 40.2, median age 39), and 74.6% in HIV-infected patients co-infected with HCV only (mean age 38.4, median age 38). Patients who were co-infected with HBV only were diagnosed with HIV at an older age (mean age 43.1, median age 41), and 70.5% were aged 20–49 years at time of HIV diagnosis.

In the HIV mono-infected group, 93.3% were men—a significantly lower proportion compared to 97.7% in those co-infected with HBV only (*p* = 0.01) (Table 1). Patients of Chinese ethnicity constituted 85.0% of those co-infected with HBV only, and this proportion was significantly higher than that of the HIV mono-infected group (76.2%) (*p* = 0.003) and the group who were co-infected with HCV only (63.2%) (*p* < 0.005). Patients of Malay ethnicity comprised 30.3% of those co-infected with HCV only, and this proportion was significantly higher than that in the HIV mono-infected group (15.1%) and in the group who were co-infected with HBV only (10.0%) (both *p* < 0.0005).

Across the four groups, the majority were infected via sexual mode of transmission (Table 1). Among patients co-infected with HCV only, 30.2% were infected through IDU—a significantly higher proportion compared to 2.2% in the HIV mono-infected group and 3.7% in those co-infected with HBV only (both *p* < 0.0005).

A significantly higher proportion of patients co-infected with HCV (40.0%) were diagnosed with HIV in the period 2006–2008, compared to 28.3% in the mono-infected group (*p* = 0.01) (Table 1). In the two groups of patients co-infected with HCV only and those with triple infection, about one-third had used recreational or illicit drugs, while the proportion with unknown history of drug use was 50% or higher. 

Among patients co-infected with HBV only, about 57.3% had both infections diagnosed within one month (Table 1). Among patients co-infected with HCV only, a significantly lower proportion of 30.2% had both infections diagnosed within one month, compared to those co-infected with HBV only (*p* < 0.0005). About one-quarter of patients co-infected with HCV only (24.9%) tested positive for HCV more than 48 months after HIV diagnosis. In the group with triple infections, about 37.5% had HIV and either HBV and/or HCV diagnosed within one month. 

**Figure 1:**
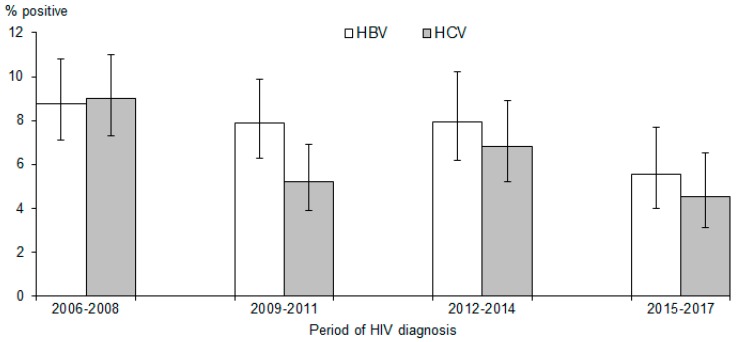
Proportions of HIV-infected patients tested positive for HBV and HCV by period of HIV diagnosis. The vertical lines indicate 95% confidence intervals.

There was a significant decline in the proportion of HIV-infected patients who were co-infected with HBV over the period of HIV diagnosis; from 8.8% in 2006–2008 (95% CI: 7.1–10.8%) to 5.5% in 2015–2017 (95% CI: 4.0–7.7%) (test for trend, *p* = 0.037) (Figure 1). The proportion of HIV-infected patients who were co-infected with HCV also saw a significant decrease; from 9.0% in 2006–2008 (95% CI: 7.3–11.0%) to 4.5% in 2015–2017 (95% CI: 3.1–6.5%) (test for trend, *p* = 0.003).

Among patients with CD4 measured at HIV diagnosis, over half (56.3%) of those co-infected with HBV only had CD4 count ≤ 200cells/mm^3^, which was significantly higher compared to 46.1% in the HIV mono-infected group (*p* = 0.005) and 39.8% in those co-infected with HCV only (*p* = 0.002) (Table 2). Among patients with aspartate aminotransferase (AST) measured at HIV diagnosis, about 26.3% of those co-infected with HBV only had AST > 48 U/liter, which was significantly higher compared to 11.6% in the HIV mono-infected group (*p* < 0.0005). Among patients with alanine aminotransferase (ALT) measured at HIV diagnosis, about 21.4% of those co-infected with HBV only had ALT > 55 U/liter, which was significantly higher compared to 10.5% in the HIV mono-infected group (*p* < 0.0005).

Of the 236 HIV patients co-infected with HBV, 216 (91.5%) underwent tenofovir-containing ART. Among the 180 patients tested positive for HBsAg who were also tested for HBeAg during the corresponding period, 85 (47.2%, 95% CI: 40.1–54.5%) were positive for HBeAg.

Univariable logistic regression analyses indicated that relative to HIV mono-infected patients, the factors associated with the presence of HBV co-infection were: Being in the age groups of 30–49 and 50–69 years, male gender, Chinese ethnicity, IDU as the mode of HIV transmission, diagnosis of HIV being made in the period 2006–2008, being diagnosed with AIDS-defining illnesses/opportunistic infections within one year of HIV diagnosis, and history of using recreational or illicit drugs (Table 3).

Multivariable logistic regression identified age group, gender, and mode of HIV transmission as significant factors that were independently associated with HBV co-infections among HIV-infected patients. Compared to patients diagnosed at 10–29 years of age, those who were diagnosed with HIV at 30–49 years (aOR 2.24, 95% CI: 1.49–3.39) and 50–69 years (aOR 2.33, 95% CI: 1.48–3.67) were at higher odds of HBV co-infections. The odds of HBV co-infections were significantly higher among men than women (aOR 2.84, 95% CI: 1.24–6.51). Compared to patients infected via sexual mode of transmission, those who were infected via IDU had over four times the odds of HBV co-infections (aOR 4.50, 95% CI: 1.41–14.38).

Factors associated with the presence of HCV co-infections in univariable logistic regression analyses were: Malay ethnicity, IDU as the mode of HIV transmission, diagnosis of HIV in the period 2006–2008, and history of using recreational or illicit drugs (Table 4). In multivariable logistic regression, ethnic group, mode of HIV transmission, and period of HIV diagnosis were found to be independently associated with HCV co-infection. Malay patients had about twice the odds of HCV infection (aOR 2.19, 95% CI: 1.10–4.34) compared with those of Indian or other ethnic minority. Compared to patients infected via sexual mode of transmission, those who were infected via IDU had nearly 20 times the odds of HCV co-infections (aOR 19.15, 95% CI: 6.74–54.38). The odds of HCV co-infections in patients diagnosed with HIV in 2006–2008 were about two times (aOR 2.00, 95% CI: 1.25–3.21) that of those diagnosed in 2015–2017.

Of the 201 HIV-infected patients co-infected with HCV within this study, 129 of them had HCV genotype testing. Excluding six with indeterminate genotype results, the common HCV genotype (GT) was GT-1 (63%), followed by GT-3 (32%) among 123 HIV-infected patients. 

The cumulative proportion of patients in the HIV mono-infected group surviving until the fifth year since HIV diagnosis was 93%, while it was 89% in those co-infected with HBV only and 91% in those co-infected with HCV only. There was no statistical difference in survival time between these three groups (log-rank test, *p* = 0.06).

## 4. Discussion

The proportions of co-infections among HIV-infected patients in our study were 7.7% for HIV-HBV co-infections, 6.0% for HIV-HCV co-infections, and 0.5% for HIV-HBV-HCV triple infections. 

The proportion of HBV co-infections in this cohort is similar to countries of low endemicity, such as the United States (7.8% to 8.6%) [20] and Germany (9.4%) [21]. In Southeast Asia, the prevalence of co-infection with HBV was estimated to be 10.4% [22]. When compared to specific neighbouring countries such as Malaysia (13%) [23], Thailand (3.3% to 13%) [24], and Indonesia (3.2% to 15.3%) [24], the proportion of HIV-HBV co-infection in Singapore is lower.

The proportion of HCV co-infection among HIV-infected patients in this study was 6.0%. In comparison, the overall prevalence of HIV-HCV co-infection in Western Europe and the United States was 25% to 30% [11] and 15.2% in Southeast Asia [22]. It is lower in Cambodia (5.5%), Myanmar (5.3%), and Thailand (5.1%) [25], while Vietnam (42.5%) and Indonesia (17.9%) have higher prevalence of co-infection [25].

The significant risk factors associated with HIV-HBV co-infection in this study were being aged 30-49 years and 50-69 years at HIV diagnosis, male gender, and HIV transmission via IDU alone (Table 3). In Southeast Asia, a higher proportion of HIV-HBV co-infections were also seen mainly in those infected via IDU. Vietnam reported a co-infection rate of 28% in people who inject drugs (PWID) [26], while 20.1% of Chinese PWID and 11.3% of Burmese PWID in the China-Myanmar border region are HIV-HBV co-infected [27]. In Western Europe and the United States, the overall prevalence of HIV-HBV co-infection in PWID was 7% to 10% [11]. Higher prevalence of HBV co-infection was also noted in HIV-positive men having sex with men (MSM) in Western Europe and the United States (9% to 17%) [11]. This was not observed among the prevalence studies done in Southeast Asia [24]. In our study, 25% of patients who acquired HIV via IDU alone were co-infected with HBV (Table 3). As the absolute number of patients infected with HIV via IDU alone or IDU and sexual transmission who were also co-infected with HBV was much smaller than those who were co-infected with HCV (10 versus 58, Table 1), this suggests that IDU contributed more to the latter group as a risk factor.

Patients with HIV-HBV co-infection were diagnosed at an older age compared to those with HIV-HCV co-infections. There was a significant decline in the proportion of patients who were co-infected with HBV over the period of HIV diagnosis, from 8.8% in 2006–2008 to 4.5% in 2015–2017. These two findings could be attributed to the introduction of hepatitis B vaccination as part of the NCIP; hepatitis B vaccination was introduced into the programme for babies born to hepatitis B-carrier mothers on 1 October 1985, and subsequently extended to all newborns on 1 September 1987 [28]. 

HIV transmission via IDU was also a significant risk factor independently associated with HIV-HCV co-infection in our study (Table 4). Our study revealed that the highest proportion of patients co-infected with HCV acquired HIV via IDU alone (56.3%), followed by those infected with HIV via IDU and sexual mode of transmission (45.8%) (Table 4). The proportion of HIV-HCV co-infection was 6.3% in patients infected with HIV via homosexual mode of transmission, 2.8% in those infected via heterosexual mode, and 7.5% in those who acquired HIV via bisexual mode (data not shown). In comparison, the prevalence of HIV-HCV co-infection in Western Europe and the United States has been reported to be affecting 72% to 95% of PWID, 1% to 12% of MSM, and 9% to 27% of heterosexuals [11]. Within the region, IDU and blood products were associated with increased risk of HIV-HCV co-infection, while female gender was a protective factor [22]. Similar high prevalence of HIV-HCV co-infections were seen in PWID: 31.8% of Chinese and 23.9% of Burmese PWID within the China-Myanmar border [27], and 89.8% to 98.5% in Vietnam [24]. As HCV is more efficiently transmitted via exposure to infected blood rather than sexual intercourse, the lower proportion of HIV-HCV co-infection in our study compared to the other European and Southeast Asia countries could be due to the relatively small number of PWID in Singapore who were infected with HIV via IDU.

Besides IDU as the mode of HIV transmission, Malay ethnicity and being diagnosed with HIV between 2006 and 2008 were also associated with HIV-HCV co-infections (Table 4). These results correspond to the pattern of drug use within Singapore. IDU is not common in Singapore, likely due to lack of supply and the deterrent effect of strict penalties for drug use and other related offences. In 2000, Subutex^®^ (or buprenorphine hydrochloride) was approved by MOH as a substitute treatment for opiate-dependent drug abusers [29]. However, it was used inappropriately and injected as an intravenous cocktail mixed with other drugs. This unexpectedly resulted in a sudden increase in the number of PWID, where at least 3800 inappropriate users of Subutex were apprehended within four years of its introduction [29]. Subsequently, Subutex was made a controlled drug on 14 August 2006 [29]. As of the end of 2017, there were no new Subutex abusers reported [30]. The episode of Subutex abuse could have explained the higher HIV-HCV co-infection rate during 2006–2008 in this study. In addition, the highest number of drug abusers were found among persons of Malay ethnicity in 2017, which could explain the association seen in our study [30].

Patients with HIV-HCV co-infection were diagnosed at a younger age compared to HIV-HBV co-infection. The younger age seen in patients infected via IDU could be explained by the pattern of drug use in Singapore: About 64% of drug abusers arrested in 2017 were below the age of 30 years [30]. 

The most common HCV genotype seen in our study was GT-1, followed by GT-3. In a study on residual blood serum samples of blood donors screened between 2011 and 2014, 0.06% were positive for HCV, and of the 42 serum samples available for genotyping, the distribution was 48% GT-3 and 31% GT-1 [31]. In Asia, GT-3 accounted for 40% of all HCV infection [32]. Regionally in Southeast Asia, GT-3 was the most common genotype seen in Thailand (44.2%) and Malaysia (58.6%), while GT-6 was more commonly seen in Cambodia (56%), Laos (95.6%), Myanmar (49%), and Vietnam (54.5%) [32]. 

There are limitations to this study. Our study is confined to patients followed-up at the NCID. However, as the NCID is the largest referral centre for HIV care, our study population could be considered as fairly representative of newly diagnosed HIV patients at large. Tests for HBV and HCV are mostly carried out on order from the treating physician, hence patients who did not have these two tests had to be excluded from the study. Treatment history was not available in the clinical database and we were unable to determine whether the HBV and/or HCV infection was chronic or acute. Patients with chronic HBV and/or HCV infection who had been tested positive more than one year prior to their HIV diagnosis could have been mis-classified as having acute infection and they were excluded from our study, but this number was expected to be small in view of the considerable proportion who would have achieved spontaneous viral clearance within six months of infection, particularly for HBV [16,17,18,19]. Moreover, all patients excluded in this way were further evaluated and found to have tested negative for HBV/HCV. Other variables such as socioeconomic status, educational level, and behavioural practices were not captured in the clinical database, but they may be potential risk factors of HBV and/or HCV co-infection. Information on other forms of hepatitis, such as hepatitis delta, was also not captured in the clinical database. 

The proportion of viral hepatitis co-infections in patients living with HIV could be higher, but as Singapore follows the United States Department of Health and Human Services (DHHS) guidelines for management of HIV, almost all patients are screened for co-infections upon HIV diagnosis [33]. As part of the local practice, patients who are found to be non-immune are usually vaccinated against HBV, hence the number of subsequent HBV infections is likely to be small, as observed in this study. On the contrary, close to 25% of co-infection of HCV was detected more than 48 months after the diagnosis of HIV (Table 1). This problem of subsequent HCV infection following HIV infection is similar to that seen in other settings, and reflects the role of ongoing high-risk sexual and drug-using behaviour in driving HIV-HCV co-infection [34]. 

Given that IDU is the only modifiable risk factor for HIV-HCV co-infection in our study, it is important to consider interventions to control the spread of infections via this route. One of the key interventions that has been implemented in the United States and Europe to reduce the risk of transmission of HIV and other blood-borne viruses among PWID is harm reduction programmes, such as needle exchange programmes. Participation in these programmes was associated with reduction in the risk of HIV incidence by 33% in the United States, and by two to three folds in Amsterdam [35,36]. The non-use of syringe exchange was associated with a six-fold increased risk of HBV and a seven-fold increased risk of HCV in the United States [37]. In Amsterdam, full participation in the harm reduction programme resulted in a six- to seven-fold reduction in the risk of HCV seroconversion [36]. In Malaysia, since the implementation of the Needle and Syringe Exchange Programme in 2006, the percentage of HIV infections acquired via IDU has reduced from 75% in 2006 to 47.7% in 2010 [38]. 

## 5. Conclusions

In conclusion, it is encouraging that there is a declining trend of co-infection with both HBV and HCV across the years, especially given that the number of new HIV diagnoses remains consistent since 2008. Many of the patients co-infected with HIV and HCV or triple infected were diagnosed with HIV at a younger age, reflecting the higher proportion of PWID seen in younger age groups. While the number of PWID in Singapore is low, it is still important to address the issue of IDU: Compared to patients infected with HIV via the sexual route, those who were infected via IDU had over four times the odds of HBV co-infections and 20 times the odds of HCV co-infections. In the absence of a HCV vaccine, other preventive interventions—such as risk-stratified screening, testing and treating, and behavioural interventions—are needed as part of hepatitis C control efforts in Singapore [39]. 

## Figures and Tables

**Table 1:** Epidemiological characteristics of HIV-infected patients with mono-infection and HBV and HCV co-infections who attended the national referral centre for HIV in Singapore, 2006–2017.

		HIV Mono-Infected	Co-Infected with HBV Only	Co-Infected with HCV Only	Co-Infected with both HBV and HCV	*p* Value ^†^	All
		*n* (%)	*n* (%)	*n* (%)	*n* (%)		*n* (%)
All		2644 (100.0)	220 (100.0)	185 (100.0)	16 (100.0)		3065 (100.0)
Age group (years) at HIV diagnosis						0.004	
	10–19	48 (1.8)	1 (0.5)	7 (3.8)	0 (0.0)		56 (1.8)
	20–29	578 (21.9)	23 (10.5)	39 (21.1)	5 (31.3)		645 (21.0)
	30–39	718 (27.2)	72 (32.7)	59 (31.9)	5 (31.3)		854 (27.9)
	40–49	654 (24.7)	60 (27.3)	40 (21.6)	4 (25.0)		758 (24.7)
	50–59	454 (17.2)	42 (19.1)	34 (18.4)	0 (0.0)		530 (17.3)
	60–69	149 (5.6)	19 (8.6)	6 (3.2)	2 (12.5)		176 (5.7)
	70+	43 (1.6)	3 (1.4)	0 (0.0)	0 (0.0)		46 (1.5)
Gender						0.032	
	Female	177 (6.7)	5 (2.3)	7 (3.8)	1 (6.3)		190 (6.2)
	Male	2467 (93.3)	215 (97.7)	178 (96.2)	15 (93.8)		2875 (93.8)
Ethnic Group						<0.0005	
	Chinese	2014 (76.2)	187 (85.0)	117 (63.2)	11 (68.8)		2329 (76.0)
	Malay	400 (15.1)	22 (10.0)	56 (30.3)	5 (31.3)		483 (15.8)
	Indian	138 (5.2)	2 (0.9)	4 (2.2)	0 (0.0)		144 (4.7)
	Others	92 (3.5)	9 (4.1)	8 (4.3)	0 (0.0)		109 (3.6)
Marital Status						0.622	
	Never married	1431 (54.1)	116 (52.7)	96 (51.9)	10 (62.5)		1653 (53.9)
	Married	591 (22.4)	48 (21.8)	32 (17.3)	3 (18.8)		674 (22.0)
	Separated/Divorced/Widowed	265 (10.0)	26 (11.8)	25 (13.5)	1 (6.3)		317 (10.3)
	Unknown	357 (13.5)	30 (13.6)	32 (17.3)	2 (12.5)		421 (13.7)
Mode of HIV transmission						<0.0005	
	Homosexual	1009 (38.2)	79 (35.9)	65 (35.1)	8 (50.0)		1161 (37.9)
	Heterosexual	1197 (45.3)	99 (45.0)	35 (18.9)	3 (18.8)		1334 (43.5)
	Bisexual	306 (11.6)	25 (11.4)	24 (13.0)	3 (18.8)		358 (11.7)
	IDU	4 (0.2)	3 (1.4)	8 (4.3)	1 (6.3)		16 (0.5)
	IDU and sexual	53 (2.0)	5 (2.3)	48 (25.9)	1 (6.3)		107 (3.5)
	Others	13 (0.5)	1 (0.5)	1 (0.5)	0 (0.0)		15 (0.5)
	Unknown	62 (2.3)	8 (3.6)	4 (2.2)	0 (0.0)		74 (2.4)
Period of HIV diagnosis						0.011	
	2006-2008	748 (28.3)	72 (32.7)	74 (40.0)	7 (43.8)		901 (29.4)
	2009-2011	755 (28.6)	63 (28.6)	40 (21.6)	5 (31.3)		863 (28.2)
	2012-2014	605 (22.9)	53 (24.1)	45 (24.3)	3 (18.8)		706 (23.0)
	2015-2017	536 (20.3)	32 (14.5)	26 (14.1)	1 (6.3)		595 (19.4)
AIDS-defining illnesses/opportunistic infections within one year of HIV diagnosis						0.113	
	No	1396 (52.8)	98 (44.5)	92 (49.7)	8 (50.0)		1594 (52.0)
	Yes	1248 (47.2)	122 (55.5)	93 (50.3)	8 (50.0)		1471 (48.0)
Ever on ART						0.620	
	Yes	2522 (95.4)	208 (94.5)	182 (98.4)	16 (100.0)		2928 (95.5)
	No	122 (4.6)	12 (5.5)	3 (1.6)	0 (0.0)		137 (4.5)
Ever used recreational or illicit drugs *						<0.0005	
	No	542 (20.5)	34 (15.5)	33 (17.8)	0 (0.0)		609 (19.9)
	Yes	251 (9.5)	27 (12.3)	61 (33.0)	6 (37.5)		345 (11.3)
	Unknown	1851 (70.0)	159 (72.3)	91 (49.2)	10 (62.5)		2111 (68.9)
Time first tested positive for HBV/HCV						<0.0005	
	Before HIV diagnosis						
	1–12 months		7 (3.2)	5 (2.7)	4 (25.0)		
	<1 month		23 (10.5)	11 (5.9)	2 (12.5)		
	From HIV diagnosis **						
	<1 month		103 (46.8)	45 (24.3)	4 (25.0)		
	1–3 months		53 (24.1)	34 (18.4)	3 (18.8)		
	4–6 months		7 (3.2)	4 (2.2)	0 (0.0)		
	7–12 months		3 (1.4)	9 (4.9)	0 (0.0)		
	13–24 months		5 (2.3)	13 (7.0)	2 (12.5)		
	25–48 months		10 (4.5)	18 (9.7)	1 (6.3)		
	>48 months		9 (4.1)	46 (24.9)	0 (0.0)		

^†^ Chi-square test was used to test for group differences. * Includes ecstasy, insufflated amyl nitrites or ‘poppers’, erectile dysfunction medications like sildenafil, amphetamines, cannabis, heroin, cocaine, barbiturates/ benzodiazepines, opium, psychedelic psilocybin mushrooms, solvents, LSD (Lysergic Acid Diethylamide). ** Includes HIV-infected patients tested positive for HBV and/or HCV on the same day.

**Table 2:** Baseline clinical characteristics of HIV-infected patients with mono-infection and HBV and HCV co-infections who attended the national referral centre for HIV in Singapore, 2006–2017 *.

		HIV Mono-Infected	Co-Infected with HBV Only	Co-Infected with HCV Only	Co-Infected with both HBV and HCV	*p* Value ^†^	All	
		*n* (%)	*n* (%)	*n* (%)	*n* (%)		*n* (%)
CD4 (cells/mm^3^); *n* (%)		2514 (100.0)	208 (100.0)	166 (100.0)	16 (100.0)	0.049	2904 (100.0)
	>350	758 (30.2)	48 (23.1)	56 (33.7)	5 (31.3)		867 (29.9)
	201–350	598 (23.8)	43 (20.7)	44 (26.5)	2 (12.5)		687 (23.7)
	≤200	1158 (46.1)	117 (56.3)	66 (39.8)	9 (56.3)		1350 (46.5)
Median CD4 [IQR]		230 [53–392]	143 [33–332]	278 [115–424]	169 [22–393]		225 [53–390]
HIV viral load (copies/mL); *n* (%)		1689 (100.0)	141 (100.0)	112 (100.0)	12 (100.0)	0.212	1954 (100.0)
	≤200	1618 (95.8)	135 (95.7)	107 (95.5)	10 (83.3)		1870 (95.7)
	>200	71 (4.2)	6 (4.3)	5 (4.5)	2 (16.7)		84 (4.3)
Median viral load in 1000 s [IQR]		83 [19–306]	176 [33–559]	83 [23–283]	74 [1–758]		87 [19–321]
AST (U/liter); *n* (%)		2021 (100.0)	179 (100.0)	125 (100.0)	12 (100.0)	<0.005	2337 (100.0)
	≤48	1787 (88.4)	132 (73.7)	101 (80.8)	8 (66.7)		2028 (86.8)
	>48	234 (11.6)	47 (26.3)	24 (19.2)	4 (33.3)		309 (13.2)
Median AST (U/liter) [IQR]		26 [21–35]	32 [27–49]	28 [23–38]	35 [29–99]		27 [22–36]
ALT (U/liter); *n* (%)		2143 (100.0)	187 (100.0)	135 (100.0)	12 (100.0)	<0.005	2477 (100.0)
	≤55	1917 (89.5)	147 (78.6)	112 (83.0)	8 (66.7)		2184 (88.2)
	>55	226 (10.5)	40 (21.4)	23 (17.0)	4 (33.3)		293 (11.8)
Median ALT (U/liter) [IQR]		24 [18–35]	30 [24–52]	25 [17–39]	36 [28–82]		24 [18–36]

^†^ Chi-square test was used to test for group differences. * Within ± 6 months of HIV diagnosis. IQR, interquartile range. ALT, alanine transaminase; AST, aspartate transaminase.

**Table 3:** Proportion and odds ratios of HBV co-infections in HIV-infected patients who attended the national referral centre for HIV in Singapore, 2006–2017.

		% Co-Infected with HBV	Univariable Model			Multivariable Model **		
			OR	(95% CI)	*p* Value	aOR	(95% CI)	*p* Value
Age at diagnosis (years)					0.001			0.001
	10–29	4.1	1.00	Referent		1.00	Referent	
	30–49	8.7	2.22	(1.47, 3.35)	<0.0005	2.24	(1.49, 3.39)	<0.0005
	50–69	8.9	2.27	(1.44, 3.57)	<0.0005	2.33	(1.48, 3.67)	<0.0005
	70+	6.5	1.62	(0.47, 5.52)	0.443	1.77	(0.52, 6.06)	0.364
Gender								
	Female	3.2	1.00	Referent		1.00	Referent	
	Male	8.0	2.67	(1.17, 6.08)	0.020	2.84	(1.24, 6.51)	0.014
Ethnic group					0.012			
	Chinese	8.5	2.04	(1.10, 3.81)	0.024			
	Malay	5.6	1.30	(0.64, 2.67)	0.471			
	Indian and others	4.3	1.00	Referent				
Marital status					0.953			
	Single	7.6	1.00	Referent				
	Married	7.6	0.99	(0.71, 1.39)	0.963			
	Divorced/Separated/Widowed	8.5	1.13	(0.73, 1.74)	0.586			
	Unknown	7.6	1.00	(0.67, 1.49)	0.988			
Mode of HIV transmission					0.067			0.047
	Sexual	7.6	1.00	Referent		1.00	Referent	
	IDU	25.0	4.05	(1.29, 12.66)	0.016	4.50	(1.41, 14.38)	0.011
	Sexual and IDU	5.6	0.72	(0.31, 1.66)	0.444	0.69	(0.30, 1.59)	0.378
	Others and unknown	10.1	1.37	(0.68, 2.76)	0.384	1.35	(0.67, 2.74)	0.404
Period of HIV diagnosis					0.148			
	2006–2008	8.8	1.64	(1.08, 2.49)	0.022			
	2009–2011	7.9	1.46	(0.95, 2.24)	0.086			
	2012–2014	7.9	1.47	(0.94, 2.29)	0.091			
	2015–2017	5.5	1.00	Referent				
AIDS-defining illnesses/opportunistic infections within one year of HIV diagnosis								
	No	6.6	1.00	Referent				
	Yes	8.8	1.36	(1.04, 1.78)	0.024			
Ever used recreational or illicit drugs *					0.057			
	No	5.6	1.00	Referent				
	Yes	9.6	1.79	(1.09, 2.94)	0.022			
	Unknown	8.0	1.47	(1.01, 2.15)	0.046			

* Includes ecstasy, insufflated amyl nitrites or ‘poppers’, erectile dysfunction medications like sildenafil, amphetamines, cannabis, heroin, cocaine, barbiturates/ benzodiazepines, opium, psychedelic psilocybin mushrooms, solvents, LSD (Lysergic Acid Diethylamide). ** Adjusted for age group, gender, and mode of HIV transmission. OR: Odds ratio. aOR: Adjusted odds ratio.

**Table 4:** Proportion and odds ratios of HCV co-infections in HIV-infected patients who attended the national referral centre for HIV in Singapore, 2006–2017.

		% Co-Infected with HCV	Univariable Model			Multivariable Model **		
			OR	(95% CI)	*p* Value	aOR	(95% CI)	*p* Value
Age at diagnosis (years)					0.800			
	10–29	7.3	1.00	Referent				
	30–49	6.7	0.92	(0.65, 1.29)	0.615			
	50–69	5.9	0.81	(0.53, 1.23)	0.317			
	70+	0.0	-	-	-	-		
Gender								
	Female	4.2	1.00	Referent				
	Male	6.7	1.64	(0.79, 3.37)	0.181			
Ethnic group					<0.0005			0.020
	Chinese	5.5	1.17	(0.64, 2.14)	0.616	1.38	(0.72, 2.62)	0.330
	Malay	12.6	2.90	(1.53, 5.50)	0.001	2.19	(1.10, 4.34)	0.026
	Indian and others	4.7	1.00	Referent		1.00	Referent	
Marital status					0.169			
	Single	6.4	1.00	Referent				
	Married	5.2	0.80	(0.54, 1.18)	0.264			
	Divorced/Separated/Widowed	8.2	1.30	(0.83, 2.04)	0.244			
	Unknown	8.1	1.28	(0.86, 1.92)	0.226			
Mode of HIV transmission					<0.0005			<0.0005
	Sexual	4.8	1.00	Referent		1.00	Referent	
	IDU	56.3	25.30	(9.28, 68.93)	<0.0005	19.15	(6.74, 54.38)	<0.0005
	Sexual and IDU	45.8	16.62	(10.95, 25.22)	<0.0005	15.01	(9.69, 23.25)	<0.0005
	Others and unknown	5.6	1.17	(0.47, 2.93)	0.736	1.10	(0.44, 2.76)	0.845
Period of HIV diagnosis					0.002			0.004
	2006–2008	9.0	2.08	(1.33, 3.25)	0.001	2.00	(1.25, 3.21)	0.004
	2009–2011	5.2	1.16	(0.71, 1.89)	0.558	1.06	(0.63, 1.77)	0.831
	2012–2014	6.8	1.53	(0.95, 2.49)	0.083	1.51	(0.91, 2.52)	0.111
	2015–2017	4.5	1.00	Referent		1.00	Referent	
AIDS-defining illnesses/opportunistic infections within one year of HIV diagnosis								
	No	6.3	1.00	Referent				
	Yes	6.9	1.10	(0.83, 1.47)	0.508			
Ever used recreational or illicit drugs *					<0.0005			
	No	5.4	1.00	Referent				
	Yes	19.4	4.21	(2.71, 6.54)	<0.0005			
	Unknown	4.8	0.88	(0.59, 1.31)	0.524			

* Includes ecstasy, insufflated amyl nitrites or ‘poppers’, erectile dysfunction medications like sildenafil, amphetamines, cannabis, heroin, cocaine, barbiturates/ benzodiazepines, opium, psychedelic psilocybin mushrooms, solvents, LSD (Lysergic Acid Diethylamide). ** Adjusted for ethnic group, mode of HIV transmission, and period of HIV diagnosis. OR: Odds ratio. aOR: Adjusted odds ratio.
